# Development of occupational health culture scale: A study based on miners and construction workers

**DOI:** 10.3389/fpubh.2022.992515

**Published:** 2022-08-22

**Authors:** Xuesong Yang, Xu Zhao, Yuhao Wang, Ruipeng Tong

**Affiliations:** School of Emergency Management and Safety Engineering, China University of Mining and Technology-Beijing, Beijing, China

**Keywords:** occupational health, scale development, culture, workplace contamination, reliability, validity

## Abstract

Culture is an essential influence on effectiveness of workplace health promotion, which can promote occupational health protection behavior. The aim of this research was to develop and validate an occupational health culture scale available to Chinese workers. Occupational health culture scale (OHCS) was developed based on elements of health culture and safety culture in workplace. Nine techniques steps of scale development were used, including a 15-member expert group, 10 workers for cognitive interview, and 1,119 questionnaires (from 710 miners and 409 construction workers) for formal investigation. Welch's variance analysis, independent samples *t*-test, Kruskal–Wallis test, Spearman correlation analysis was employed, respectively, to verified nine hypotheses about impact relationship on OHCS score. After the analysis reliability and validity, the final scale consisted of 21 items in five domains: leadership support, co-workers support, values, policy and norms, employee involvement, physical environment. Moreover, respirable dust concentration from individual sampler had the largest negative correlation coefficient on OHCS score, −0.469 (*p* < 0.01). The development of an occupational health culture among Chinese workers is necessary for the sustainability of human resources and the implementation of corporate responsibility.

## Introduction

With 160 million new cases of work-related diseases worldwide each year, the long-term loss of human resources from unhealthy workplaces is a daunting challenge for governments (1). Hazards generated in the production process, such as dust, noise, volatile organic compounds (VOCs), is the focus of cleaner production, resulting in the burden of disease and economic losses (2). Long-term occupational exposures may develop many diseases, especially those caused by respiratory exposure to occupational hazards, have a high correlation with attributable deaths (3). The burden of disease from occupational carcinogens in China increased significantly from 1990 to 2017 (4). Even if workers release their jobs, they still have a high occupational health risk because the health damage of occupational health hazards to workers is chronic and cumulative (5, 6). If there is the lack of effective health protection, and that would cause long-term, irreversible health damage to workers. The management of occupational health is carried out within the framework of the occupational health and safety management system (OHSMS). Because of the non-specific nature of OHSMSs, vague definition and wide range of applications, there are no operational measures in the actual management of occupational health (7). The occupational health management component of the OHSMS, however, seems to have stagnated (8). Also, for the health of worker, employer support is needed to complete the implementation of worker health monitoring, but most employers are not positive toward workers' health surveillance (WHS) (9).

Workplace is considered an excellent place for health promotion because employees work almost half of the entire life, which is a good place for preventing chronic diseases, mental illnesses, and improving unhealthy lifestyles (10). Advocacy of health-protective behaviors and health interventions in the workplace further influence families and communities by employees and employers, creating an attendant effect that reduces health care costs for society as well as fulfilling corporate social responsibility (CSR) (11, 12). Culture is an essential influence on the implementation of health promotion initiatives, and a supportive culture can increase the effectiveness of workplace health promotion, particularly in achieving lasting goals of reducing population health risks (13, 14). Culture is an abstract concept, and research focus on its influence on values, attitudes, beliefs, and behaviors, among other things, and so it is with culture of health. Organizational culture is the shared values, beliefs or perceptions held by employees within an organization, and is necessary for the future competition of the industries which have a dominant risk-averse mindset, like mining industry (15, 16). Evidence suggests that health promotion programs that incorporate more cultural elements into their strategies reduce health risks of employee by 5% per year, which is 2.5 times higher than health promotion programs that do not include cultural elements (17). Purposeful design is a crucial point to creating and maintaining a healthy workplace culture (18). If a conception of occupational health culture (OHC) was proposed, there should be clarification of the vision and the elements.

For workplaces with high health risk, in addition to mandatory occupational health precautions by employers, some other interventions should be considered to reduce the health damage caused by occupational hazards. If protective behaviors of occupational health are expected to be reinforced, OHC is inevitably an intervention way. Therefore, the purpose of this study is to develop and validate an occupational health culture scale (OHCS), which to quantify individual-level scores on perceptions of organization's OHC and preliminarily explore factors influencing individual OHCS perception scores.

## Literature review

### Organizational health culture

There are many definitions of organizational culture or corporate culture, Schein (19, 20) distinguishes basic levels of organizational culture, observable artifacts, values, basic underlying assumptions, culture that can be studied espoused, and documented culture elements through investigation. Thus, organizational culture is expressed through the shared values, beliefs, or perceptions held by employees within an organization or organizational unit (15). Culture is formed spontaneously among people and so do in organizations, with internal stability and a tendency to change (21, 22). Organizational culture is considered to be the “glue or a linking pin” between people and the organization, as well as a phenomenon that reflects the learning processes, and activities of competence building are phenomena (23). It has a positive tendency to change employee behavior, especially health and safety behavior, and is effective in various organizational accidents (15, 24, 25). A workplace health culture, which can be viewed as the wellness culture of the organization to which it belongs, is a sub-aspect, or sub-culture, of wellness, and it is in the interest of the employer to foster a sub-culture that promotes wellness (26). Therefore, the safety culture and health culture of the organization are developed as subcultures in the organization.

Research on the culture of health in the workplace began earlier in developed economies and is still dominating, especially in North America and Europe. The Health Enhancement Research Organization (HERO) established the Culture of Health Research Committee in 2013 in the United States to identify key elements and conceptual frameworks for a culture of health, defining a list of 24 elements that are relevant to all types of workplaces (18). Golaszewski et al. (27) developed a workplace health culture scale based on Allen's Lifegain Health Culture Audit (LHCA) to assess the impact of health culture in five dimensions (28, 29). Aldana et al. (30), on the basis of the HERO Scorecard, argued that culture change needs to launch in five aspects. The CDC viewed a culture of health as a work environment that promotes employee safety and health, without mentioning the processes and mechanisms by which the environment affects employees, which is precisely the important part of a workplace culture of health (13, 31). Safeer and Allen (13) summarized six primary and overlapping spheres of culture influence, incorporating lists presented by the Wellness Councils of America, the Health Enhancement Research Organization (HERO), and the CDC. Researchers have almost always focused on elements such as values, norms, and climate, with adaptations in presentation. Kent et al. (32) and Kwon et al. (33) focused on the distinction between the influence of top leaders, managers, and individuals, arguing that the key elements that promote a healthy culture are leadership commitment, social and physical environmental support, and employee engagement.

### Organizational safety culture

Safety culture has been mentioned in the course of research on organizational health culture, for example, paying attention to what can be learned from safety culture, which has been conducted in a more mature and systematic manner (34, 35). Some of the researches on the definition of safety culture included health culture (36, 37); there are also definitions of health culture that suggested that safety culture as a component of health culture may be a fruitful direction for research (13, 38). As can be seen, safety culture and health culture, in organizations, appear to be two parallel subcomponents of organizational culture and are two overlapping and controversial concepts. In some high-risk industries, safety culture (or health culture) completely dominates the organizational culture, but cannot be separated from it, as there are similar concepts and both focus on the way people think and behave in terms of safety (or health) to varying degrees (38). A people-centric culture enhanced organizational safety maturity, leading to brilliant safety performance (39).

However, few studies have been conducted on special workplace that specialize in health culture or health promotion. On the contrary, there is a lot of research on safety culture in high-risk workplace, focusing on accident prevention because safety culture was considered as an effective means to prevent accidents (36, 40–42). Therefore, in designing an OHC, it is necessary to make reference to safety culture. Fu's (43) team added seven elements to the 25 safety culture indicators proposed by Stewart (44) to form a 32-element safety culture measurement scale, and the content of the safety culture was significantly modified to fit the Chinese cultural context.

### Occupational health culture

In China, some researchers have tried to develop workplace health culture scales, for example, Chang et al. (34, 45) developed the workplace health culture scale in Taiwan and has incorporated it into the implementation of workplace health promotion; Jia et al. (46, 47) has also developed a workplace health culture scale for China, showing application of it. However, little attention has been paid to current workplace health culture research for workers exposed to occupational hazards, such as dust, VOCs, noise. A culture of health is a key element in the success of health promotion implementation, and the prevention of occupational diseases should be considered in the context of health promotion (32). For workers exposed to well-defined occupational hazards, especially in developing countries, it is necessary to develop OHC that can avoid health damage. China is currently experiencing a dramatic increase in the number of patients with occupational-related diseases.

On the basis of Jia et al. (48) and Chang et al. (34) who developed workplace culture scales in the Chinese, this paper considered the “shared beliefs and behaviors,” the core of the culture, emphasized by Safeer and Allen (13). In addition, research of Fu (43) on occupational safety culture in China will be integrated into this OHC. Because, according to Fu's conclusions, it can be argued that an excellent OHC can influence an organization to pay attention to employees' occupational health, establish a sound occupational health management system, and then reduce employees' health risks in terms of technologies, employee health management, and financial investment. Since 2007, Fu' team have conducted a survey of 82 companies in China with 4,368 employees to measure the safety culture of the company (49). There are also many similarities between Fu's safety culture elements and the Culture of Health Research Committee's health culture elements, such as both mentioning participation, communication, training, as shown in the Supplementary Table 1. Therefore, our OHCS not only draws from the Culture of Health Research Committee's workplace health culture elements, but also considers the safety culture elements proposed by Fu.

The workplace OHC we initiated is different from other studies that called “workplace culture of health (CoH),” “workplace health culture,” and “healthy worksite culture.” However, the core ideas and essence are similar. OHC in this study is a directive to occupational health promotion for numerous existences of occupational hazards, which corresponds to China's current requirements. Through literature analysis, six domains were generated as following: occupational health (1) leadership support (OHLS), (2) co-workers support (OHCWS), (3) values (OHV), (5) policy and norms (OHPN), (6) employee involvement (OHEI), (7) physical environment (OHPE). The definitions or applications of each domain were found in the literatures (13, 27, 30, 32–34, 50, 51), and then we created preliminary OHC definitions by deductive and inductive methods as shown in Supplementary Table 2.

## Materials and methods

**Figure 1:**
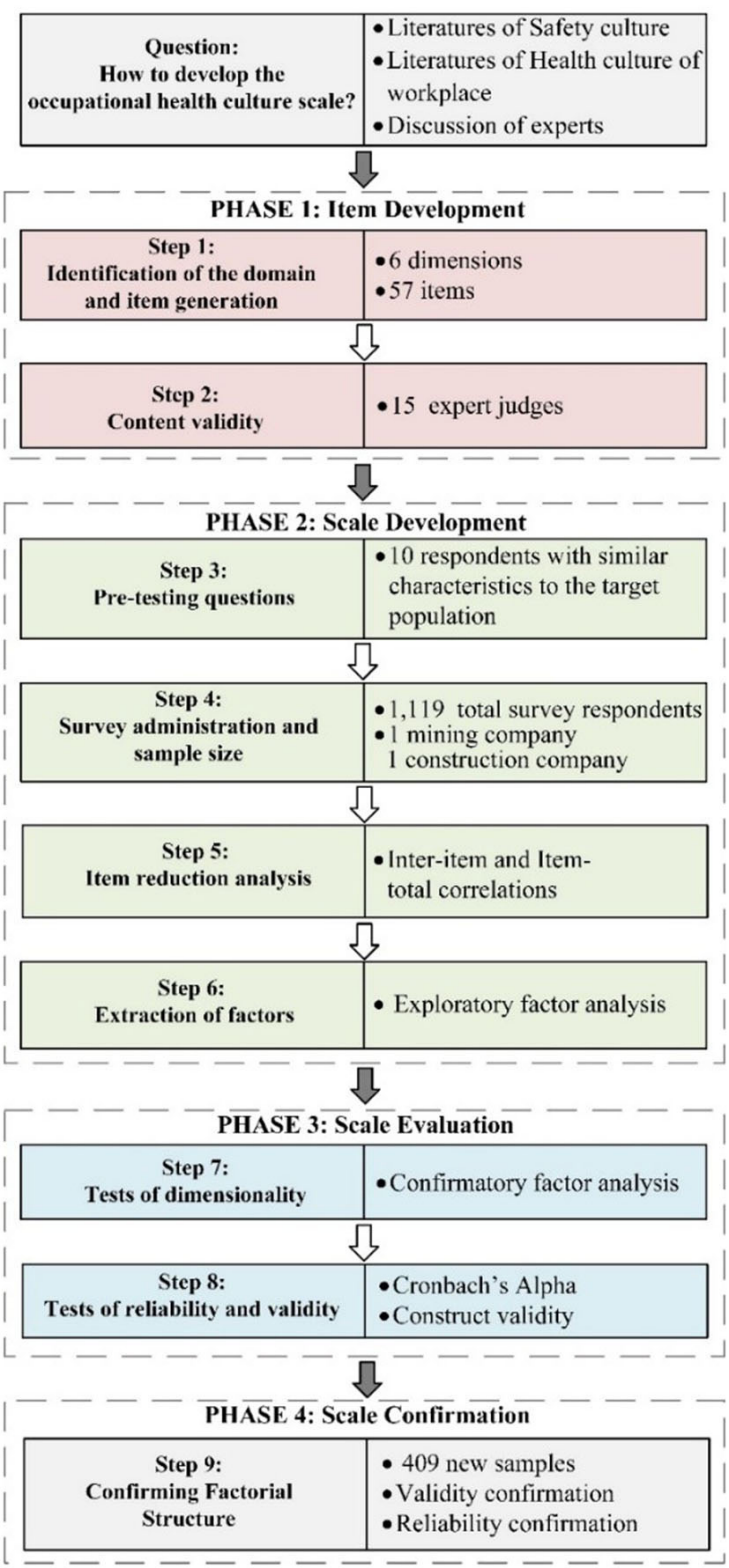
The technical flowchart of scale development of occupational health culture.

For the OHCS development technique, we learned from Boateng et al. (52) for the nine steps of the scale development practices, and also used the common methods in item generation, theoretical analysis, and psychological analysis as reviewed by Morgado et al. (53). In addition, we selected workers investigated from two different organizations for pre-test, evaluation and confirmation. The technical flowchart of scale development for this study is shown in the Figure 1.

### Design of the scale

#### Generation of items

Fifty-seven items were initially designed. All of items reflect the cognitive level of OHC through the employee's perceptions. The subject of the items is usually expressed as “I” or “We,” and “We” refers to “I and my co-workers.” A 5-point Likert scale was used to assign and score each item, named from “strongly disagree” to “strongly agree.” In these six domains: there were 11 items in OHLS, containing two aspects, senior leaders and supervisors, leadership commitment, leadership responsibility, and supervisors' interaction with workers; OHCWS contained nine items, representing attitudes, assistance, and supervision of occupational health promotion among workers; OHV contained eight items, consisting of shared values and personal values; OHPN contained 12 items, including employees' formal or informal attitudes and behaviors toward the company's occupational health policies, management, systems; OHEI contained nine items, including engagement in cleaner production programs, promotion activities, utilization healthy resources; there were eight items available in OHPE, representing employee views on occupational hazards in the work environment and protection against them (Supplementary Table 3).

#### Optimization of items

We established a group of fifteen experts including health promotion, occupational disease prevention, safety culture with experience in scale development, industrial hygiene engineers, occupational health physician, OHS managers with more than 10 years of experience. Experts were asked to test the content validity of our initial items. And then, in pre-test phase, we used the cognitive interview method, which is also the recommended method for scale development (52, 54). We randomly selected 10 workers from the target sample, discussed the preliminary scale with them. In this process, our researchers followed the whole course of answering the questions and recorded respondents' experience to obtain their thoughts about answering the questions and suggestions for the items. This process was similar to a semi-structured interview.

### Samples

Mining and construction industries were selected by this paper, because they are the industries of concern in China with serious occupational health hazards (55, 56). And also, our research team has long-term and deep research cooperation about OHS project with some large companies in these two industries. In 2020, the research team conducted a project called “Innovation Program for Occupational Safety and Health Management Model” with a large mining company in China. And in 2021, the research team launched a project called “Standardization of Occupational Health Management in Construction Projects” with a regional company of a large real estate group in China. As a result of the support, the respondents were from these two companies. There are two studies (study 1 and study 2) in this paper were carried out for development and validation of the scale. The company in study 1 was a copper mine located in northwest China, where 750 questionnaires were distributed on site and 732 questionnaires were collected, with 710 valid questionnaires. Study 2 was conducted in a construction company located in southwest China. We returned 438 questionnaires from 450 ones, but 409 questionnaires were completed.

### Statistical analysis

Prior to factor analysis, inter-item and item-total correlations were estimated for all items, a common technique used to support item deletion or modification. This is the domain of classical test theory (CTT) (52).

#### Factor analysis

In the exploratory factor analysis, principal component factor analysis with the varimax rotation eigenvalue criterion >1.0 were employed detect the latent variable. Before identifying the latent variables, we confirmed the feasibility of factor analysis by using Kaiser-Meyer-Olkin (KMO) test and Bartlett's sphericity test. Usually, KMO > 0.8 and significance (*P*-value) of Bartlett's sphericity test <0.01 are suitable for factor analysis. Next, the factor loadings and cross-loading of items were obtained by component matrix after rotation. Items with factor loadings >0.5 and cross-loadings <0.5 were considered (57). Each potential factor should contain more than three items.

After completing the exploratory factor analysis, a conceptual model was constructed and a confirmation factor analysis was performed for factor structure. The factors should be considered as latent variables of common influence with each other.

We identified and confirmed the model fit by the absolute fit index and the relative fit index. Absolute fit index contains: Chi-square/degree of freedom (χ^2^/df), goodness-of-fit index (GFI), adjusted for the model's degrees of freedom (AGFI), root mean square error of approximation (RMSEA), standardized root mean square residual (SRMR). And relative fit index contains: normal fit index (NFI), incremental fit index (IFI), Tucker-Lewis Index (TLI), comparative fit index (CFI). Their criterions are available in Supplementary Table 4. Both IBM SPSS 24.0 and IBM AMOS 25.0 were used to complete the factor analysis. In addition, the convergent validity is expressed by the average variance extracted (AVE), and AVE > 0.5 indicates acceptable convergence. The Cronbach's alpha coefficient is usually used to characterize internal consistency of the scale items, with a value between 0 and 1, and the larger the value, the higher the reliability, which 0.70 is as an acceptable threshold. The composite reliability (CR) value >0.70 represents an appropriate construct reliability.

#### Confirmation of the validity and reliability of the OHCS

Furthermore, factor structure developed through the factor analysis in study 1 was used to confirm the reliability and validity of the upgraded scale. Therefore, study 2 was initiated and we selected another sample from company B (a construction company) with 409 construction workers to complete the OHCS. In study 2, absolute fit index, relative fit index, AVE, Cronbach's α, and CR were analyzed once more.

### Correlation analysis between OHC and personal factor

**Figure 2:**
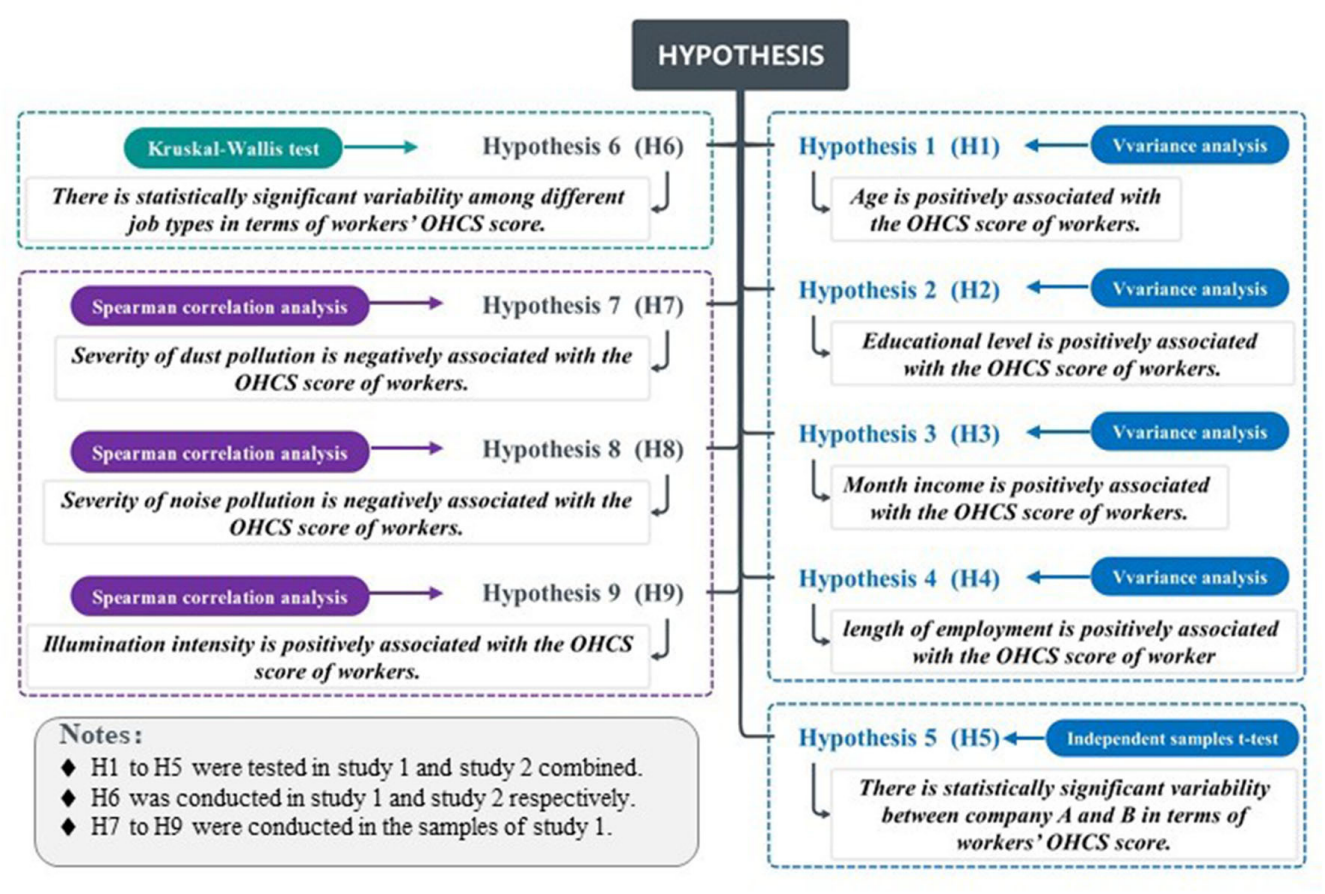
Hypothesis for correlation between OHCS scores and personal factor.

We proposed nine hypotheses and test whether there were significant differences in the demographic characteristics of the respondents' perceptions of OHC in study 1 and study 2 (Figure 2). To validate H7 to H9, we consulted inspection reports of occupational hazards for the copper mine from 2015 to 2020. For occupational hazards exposure amount, we used time-weighted averages for comparative analysis because underground workers in different jobs spend different amounts of time. For dust concentration, we obtained total dust, respiratory dust measured at fixed monitoring points and respiratory dust measured by individual samplers. Noise analyzer measure the noise value at fixed measure point of workplace, and illumination intensity measured by illuminance meter at fixed monitoring points. IBM SPSS 24.0 software was used to perform the operations of hypothesis testing. The monitoring data was measured and reported by independent third-party organizations commissioned by the mine, which are occupational health service providers and have official certification in China. Occupational hazards are detected and analyzed using the corresponding Chinese official standard, as following:

“Determination of dust in the air of workplace Part 1: Total dust concentration [GBZ/T 192.1-2007]”, “Determination of dust in the air of workplace Part 2: Respirable dust concentration [GBZ/T 192.2-2007]”, “Measurement of Physical Agents in Workplace Part 8: Noise [GBZ/T189.8-2007]”, “Measurement methods for lighting [GB/T 5700-2008]”.

The inspecting period for the data used in this research was the early August for each year from 2015 to 2020, obtained from the mining company files. The inspection results could not all be matched to our OHCS respondents. We selected eight workplaces: explosives magazine duty room, ore porter duty room, crushing chamber, belt station, filling station, support worksite, machine shop, and drilling worksite. In addition, it is assumed that the jobs in these eight sites are explosive magazine administrators, ore porters, crushers, transport belt miners, filling workers, support miners, machine repairers, rock drillers. Data from measurement points were selected for each workplace, and were averaged over the 6 years from 2015 to 2020.

## Results

### Content validity and pre-testing

The content validity test and pretest were conducted simultaneously. In the content validity assessment, eight items were removed according to the experts' consistent recommendations and expert voting, all of which were of poor fitness with the scale objectives, less importance, and strong similarity with other items. In addition, through the cognitive interview, we modified the items with poor representation, using a formulation more conducive to workers' understanding, without changing the content of items. We also removed three items that most workers (eight out of ten) agreed were invalid, or items that were difficult to answer. Finally, six domains with a total of 46 items were identified (as shown in Supplementary Table 5).

### Demographics

In study 1, the respondents were underground frontline miners, and therefore, all of them was male. Fifteen underground job types were included, and Table 1 shows the demographic information of the miner respondents in study 1. Table 1 also shows the demographic information of 409 construction workers in study 2, which also contains the job types, age, education, monthly income, and length of work.

**Table 1:** Demographic information of the respondents in study 1 and study 2.

**Variable**	**Study 1: Number (Proportion, %)**	**Study 2: Number (Proportion, %)**	**Variable**	**Study 1: Number (Proportion, %)**	**Study 2: Number (Proportion, %)**
**Age**			**Job type of miners**		
18 ~ 30	9 (1.27)	5 (1.22)	Safety officer (SO)	38 (5.35)	–
31 ~ 40	231 (32.54)	53 (12.96)	Foreman	62 (8.73)	–
41 ~ 50	394 (55.49)	185 (45.23)	Blaster	21 (2.96)	–
51 ~ 60	75 (10.56)	132 (32.27)	Filling worker (FW)	105 (14.79)	–
>60	1 (0.14)	34 (8.31)	Ore porter (OP)	93 (13.10)	–
**Education level**			Electric locomotive operator (ELO)	8 (1.13)	–
Primary school or below	12 (1.69)	–	Loading miners (LM)	18 (2.54)	–
Junior high school	298 (41.97)	122 (29.83)	Machine repairer (MR)	18 (2.54)	–
High school or technical secondary school	286 (40.28)	235 (57.46)	Hoist engine operator (HEO)	8 (1.13)	–
Junior college	106 (14.93)	49 (11.98)	Transport belt miner (TBM)	34 (4.79)	–
Bachelor or above	8 (1.13)	3 (0.73)	Crusher	78 (10.99)	–
**Month income (RMB)**			Signaling miner (SM)	30 (4.23)	–
<5,000	–	–	Rock driller (RD)	134 (18.87)	–
5,000 ~ 7,000	–	13 (3.81)	Explosive magazine administrator (EMA)	10 (1.41)	–
7,000 ~ 9,000	114 (16.06)	154 (37.65)	Support miner (SUM)	53 (7.47)	–
9,000 ~ 11,000	312 (43.94)	153 (37.41)	**Job type of construction workers**		
>11,000	284 (40)	89 (21.76)	Polisher	–	35 (8.56)
**Length of work (year)**			Electric welder (EW)	–	38 (9.29)
<5	109 (15.35)	17 (4.16)	Plumber	–	74 (18.09)
6 ~ 10	319 (44.93)	82 (20.05)	Concrete worker (COW)	–	56 (13.69)
11 ~ 15	256 (36.06)	259 (63.33)	Equipment installer (EQI)	–	35 (8.56)
16 ~ 20	24 (3.38)	51 (12.47)	Scaffolder	–	33 (8.07)
>20	2 (0.28)	–	Carpentry	–	34 (8.31)
			Bricklayer	–	49 (11.98)
			Stoneworker	–	10 (2.45)
			Decoration workers (DW)	–	45 (11)

The classification of job types in our study is mainly based on the staffing table of labor positions in the two companies. Information on copper mine jobs comes from company documents, as well as from local standards or industry standards in China. The job classification of construction workers mainly comes from the catalog of job types in the housing and urban-rural construction industry issued by the Chinese Ministry of Housing and Urban-Rural Development of the People's Republic of China.

### Reliability and exploratory factor analysis

The first exploratory factor analysis yielded 12 latent variables with eigenvalues >1, accounting for 73.9% of the total variance. The KMO test for sampling adequacy was 0.924, and the Bartlett test for sphericity was highly significant (*p* < 0.001). By observing the inter-item and item-total correlations of forty-six items, we eliminated items with correlation coefficients <0.5. Therefore, twenty-two items with poor inter-item and item-total correlations were eliminated, in which the items in OHCWS were significantly uncorrelated with the performance of other dimensional items, so the domain of co-worker support was removed. In the factor loadings and cross-loadings, one item in OHPE, one item in OHEI, and three items in PN formed a latent variable which the factor loadings were >0.5, but with poor inter-item and item-total correlations, thereby removing this factor. Finally, twenty-four items with five domains remained, with a total variance of 81.92%.

### Confirmatory factor analysis

#### Confirmation factor analysis of study 1

**Figure 3:**
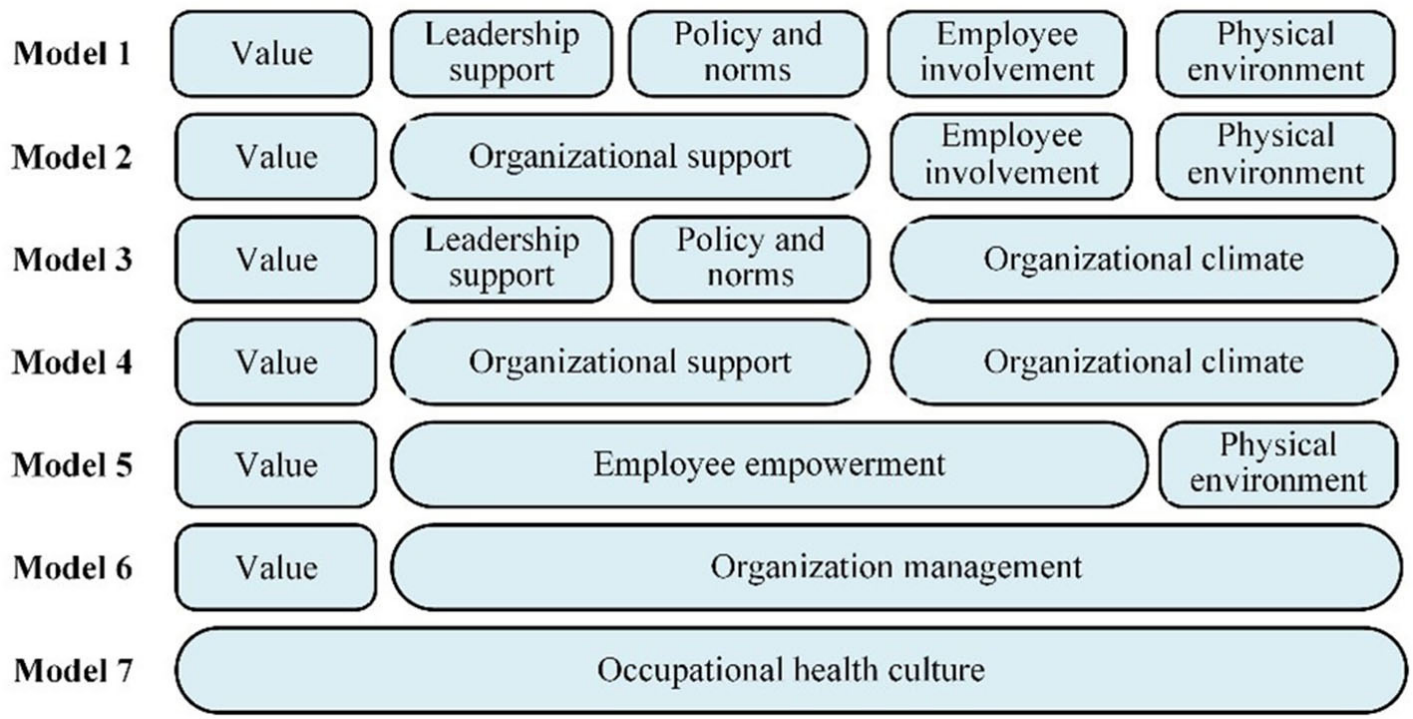
Seven factor model occupational health culture.

Confirmatory factor analysis was employed to analyze the degree of fit between the observed data and the conceptual model according to the respondent data from study 1. Specifically, the invisible latent variables obtained by observing the Likert scores of each item in the screened scale with five domains, namely, OHLS, OHV, OHPN, OHEI, OHPE. In order to verify the rationality and accuracy of this five-factor model, we designed six hypothetical competing models to compare with our ideal model (58), as shown in Figure 3. The results showed that the fit of the five-factor model is much better than the other models, as shown in the Table 2. The fit indexes of five-factor model were acceptable.

**Table 2:** Main fitness indicators of the OHC factor model.

**Models**	**χ^2^**	**df**	**χ^2^/df**	**GFI**	**AGFI**	**NFI**	**IFI**	**TLI**	**CFI**	**RMSEA**	**SRMR**
Model 1	1,035.379	242	4.278	0.889	0.863	0.944	0.956	0.95	0.936	0.068	0.0371
Model 2	4,308.403	246	17.514	0.592	0.503	0.766	0.777	0.749	0.776	0.153	0.1111
Model 3	3,408.788	246	13.857	0.672	0.601	0.815	0.826	0.805	0.826	0.135	0.1104
Model 4	6,676.347	249	26.813	0.489	0.384	0.638	0.647	0.608	0.646	0.191	0.1544
Model 5	6,483.527	249	26.038	0.481	0.375	0.648	0.657	0.62	0.657	0.188	0.1332
Model 6	8,338.897	251	33.223	0.401	0.285	0.548	0.555	0.511	0.555	0.213	0.1307
Model 7	9,909.91	252	39.325	0.369	0.249	0.463	0.469	0.418	0.468	0.232	0.1396
Modified model 1	657.093	179	3.671	0.918	0.894	0.959	0.97	0.964	0.97	0.061	0.0342

We modified the five-factor model according to modification index (M.I.) value. Item OHV5, OHPN3, and OHPE5, were excluded after three times of correction, until the fitting indexes did not improve significantly, as shown in Table 2. The ultimate questionnaire containing 21 items was proposed (as shown in Supplementary Table 6). For modified models, the Cronbach alpha coefficient and CR of each factor exceeded the threshold of 0.70, and the corrected item-to-total correlations were all >0.5, meaning good internal consistency reliability (Table 3). The square root of AVE exceeds the factor correlation, and thus presenting great convergent and discriminant validity, as shown in the Table 4.

**Table 3:** Factor loading results for the 21 items in study 1.

**Domains**	**Items**	**Corrected item-to-total correlation**	**Cronbach's α**	**Factor loadings**					**CR**	**AVE**	**Total explained variance (%)**
				**1**	**2**	**3**	**4**	**5**			
Leadership support	LS3	0.658	0.949	0.883					0.929	0.726	20.48
	LS4	0.693		0.872							
	LS8	0.700		0.878							
	LS9	0.683		0.897							
	LS10	0.584		0.716							
Values	V1	0.653	0.945		0.86				0.883	0.715	12.53
	V4	0.662			0.845						
	V7	0.690			0.832						
Policy and norms	PN4	0.717	0.943			0.839			0.916	0.685	19.49
	PN5	0.623				0.858					
	PN8	0.661				0.769					
	PN9	0.742				0.814					
	PN11	0.723				0.854					
Employee involvement	EI5	0.583	0.941				0.843		0.921	0.744	16.6
	EI6	0.603					0.888				
	EI7	0.685					0.856				
	EI9	0.593					0.863				
Physical environment	PE1	0.548	0.901					0.729	0.869	0.628	14.51
	PE4	0.660						0.738			
	PE6	0.690						0.836			
	PE7	0.694						0.851			

**Table 4:** Correlations between factor structures in study 1.

	**OHV**	**OHLS**	**OHPN**	**OHEI**	**OHPE**
OHV	–				
OHLS	0.399^***^	–			
OHPN	0.594^***^	0.52^***^	–		
OHEI	0.52^***^	0.433^***^	0.486^***^	–	
OHPE	0.532^***^	0.564^***^	0.5^***^	0.43^***^	–
Square root of AVE	0.846	0.852	0.828	0.863	0.792

*** P < 0.001.

#### Confirmation factor analysis of study 2

In study 2, we revalidated the reliability and validity of the scale, the relevant data of which are presented in the Supplementary Tables 7, 8. Great convergent and discriminant validity were also available in study 2. The CR value of each factor was >0.8, and the Cronbach's alpha coefficient was 0.952, which proved to have good internal consistency. In addition, OHCS had satisfactory fit indexes in study 2: χ^2^/df = 3.525, NFI = 0.931, IFI = 0.95, TLI = 0.941, CFI = 0.949, RMSEA = 0.079, and SRMR = 0.0393, thus confirming the construct validity of the scale.

### Hypothetical test

By conducting test of variance on the two samples, it was found that the group for age, education level, month income, and length of work did not satisfy the assumption of homogeneity, Welch's variance analysis and Tamhanes *post-hoc* comparison were used. The results of the differences in individual demographic characteristics of the OHCS scores of 1,119 respondents are shown in the Supplementary Table 9 and were used to test hypotheses H1–H4.

**Figure 4:**
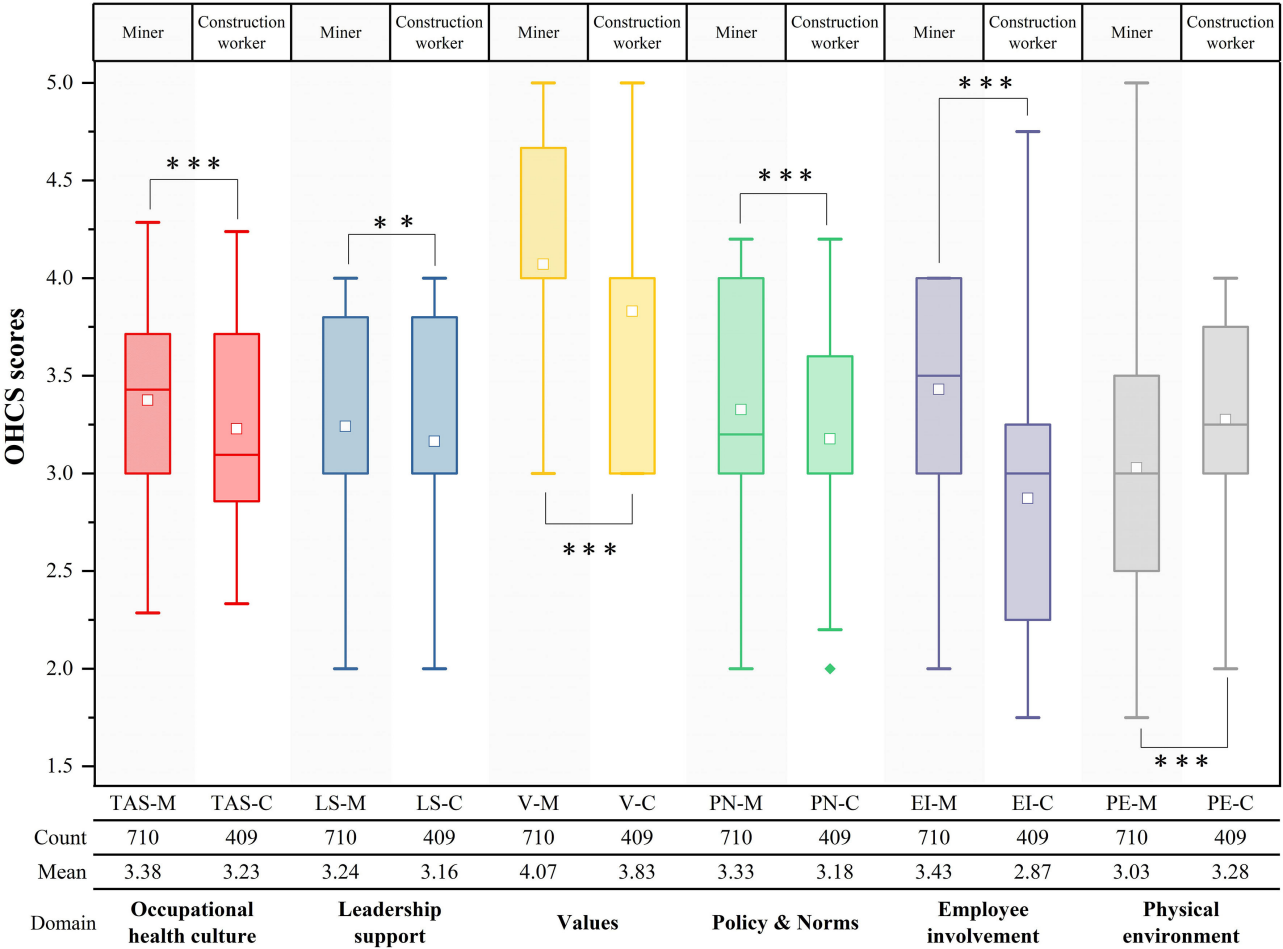
Analysis of the difference in OHCS scores between miners and construction workers. TAS-M, total average occupational health scale scores of miners; TAS-C, total average occupational health scale scores of construction workers. ***P < 0.001, **P < 0.01.

**Figure 5:**
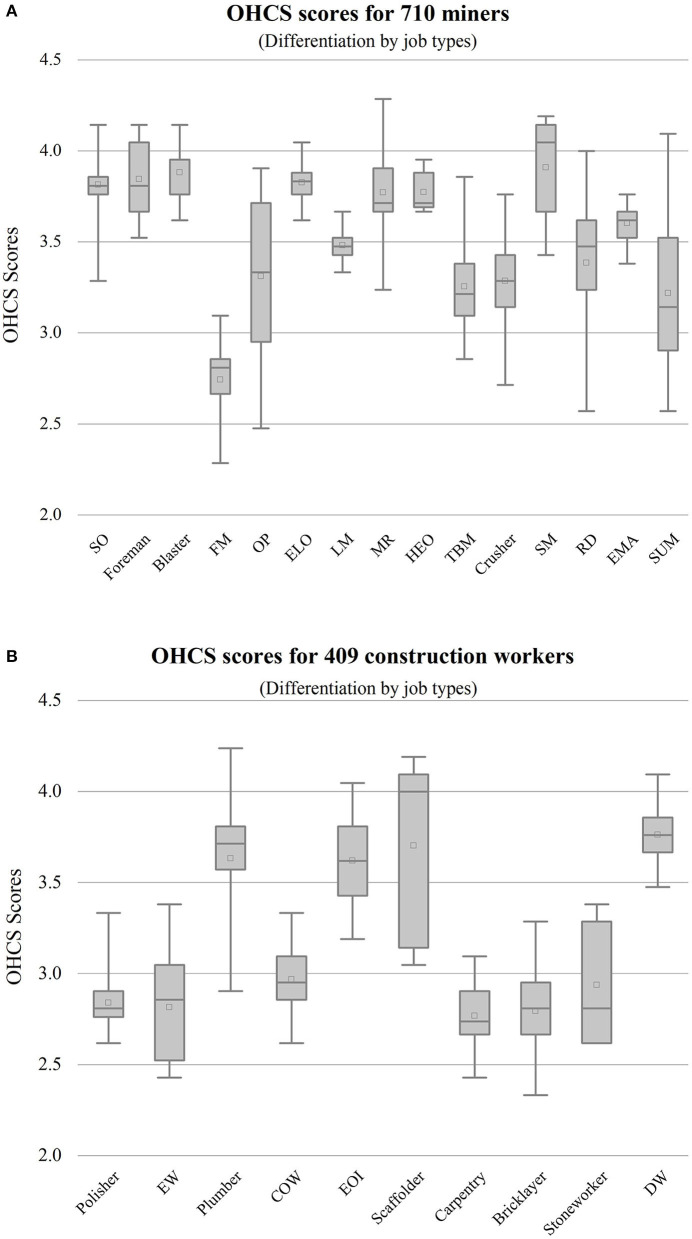
**(A)** OHCS score distribution of miners. SO, safety officer; FM, filling worker; OP, ore porter; ELO, electric locomotive operator; LM, loading miners; MR, machine repairer; HEO, hoist engine operator; TBM, transport belt miner; SM, signaling miner; RD, rock driller; EMA, explosive magazine administrator; SUM, support miner. **(B)** OHCS score distribution of construction workers. EW, electric welder; COW, concrete worker; EOI, equipment installer; DW, decoration workers.

The positive association between higher age group and OHCS scores was not significant, but rather the OHCS scores of respondents in the two groups aged 18–30 and 60 years were higher than those aged 31–60 years. There were significant positive association of higher education level and higher month income with OHCS scores in the results. Moreover, the significance of the positive correlation between higher length of work on OHCS scores was not obvious. Hypothesis H2, H3, H4 were confirmed. An independent samples *t*-test of the OHC scores of study 1 and study 2, that were, OHCS scores of miners and construction workers, revealed that there were significant differences in OHCS scores of miners and construction workers in terms of OHCS mean scores, supporting hypothesis H5, as shown in Figure 4. The Kruskal-Wallis test for the fifteen job types in study 1 showed significant results (*p* < 0.001), indicating that the OHCS scores were significantly different among the miner job types (Figure 5A). Similarly, we conducted Kruskal–Wallis tests for the ten job types in study 2 and the results were significant (*p* < 0.001), as shown in Figure 5B, which confirmed the hypothesis H6.

**Figure 6:**
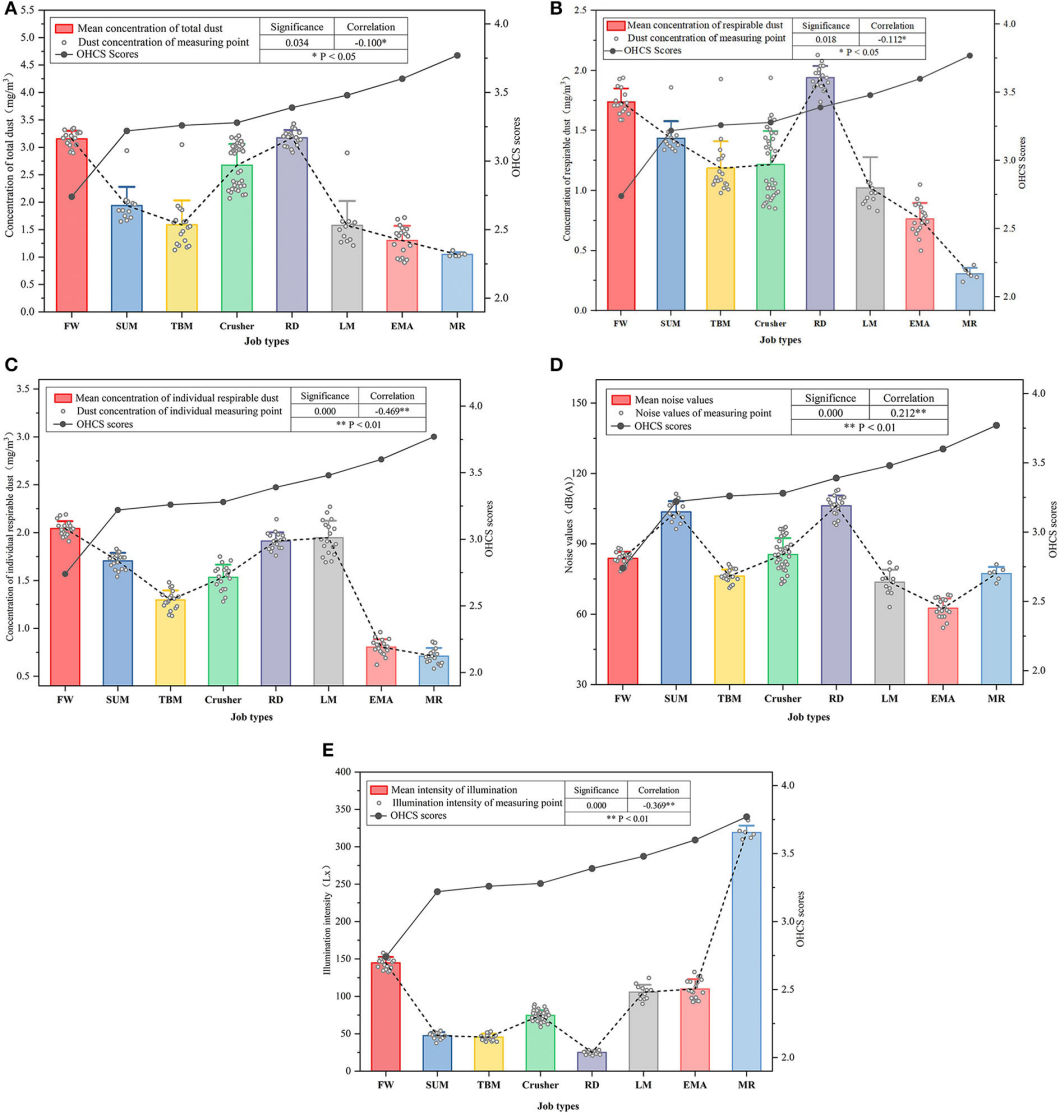
**(A)** Workplace total dust concentration and miners' OHCS score distribution. **(B)** Workplace respirable dust concentration and miners' OHCS score distribution. **(C)** Workplace individual respirable dust concentration and miners' OHCS score. **(D)** Workplace noise values and miners' OHCS score distribution. **(E)** Workplace illumination and miners' OHCS score distribution.

The OHCS score was significantly correlated with exposure to dust, noise, and illumination by Spearman correlation analysis (*p* < 0.05), shown as Supplementary Table 10. Figures 6A–E shows the distribution of OHCS scores and exposure of total dust, respirable dust, individual respirable dust, noise value, and illumination as well as their correlation. Although miners' OHCS scores statistically negatively associated with exposure concentration of total dust and respiratory dust, the correlation coefficients were relatively low, as shown in Figures 6A,B. The highest correlation coefficient between respiratory dust concentration obtained from individual sampler and OHCS score, supported hypothesis H7 (Figure 6C). The correlation between noise values and OHCS scores was 0.212 (*p* < 0.01), indicating that the noisier the workplace, the higher the workers' OHCS scores are likely to be, so hypothesis H8 was not valid (Figure 6D). It is noteworthy that illumination has a significant negative correlation with OHCS score with a correlation coefficient of −0.369 (*p* < 0.01), so hypothesis H9 was also not valid (Figure 6E).

## Discussion

### Explanation of this scale

Currently there are two ways of developing workplace health culture scales: 1) to confirm the reliability and validity of the scale by a large survey sample (*N* > 2,000), discriminant validity among several organizations was tested (34), or to confirm the criterion validity of the newly developed scale through another similar and developed scale (27); the other is Kwon's method containing two studies that the first study to assess the reliability and validity of the scale, and the second study to confirm the reliability and validity of final scale (33). For this paper, we mainly refer to Kwon's method, that is, dividing the two samples into study 1 (*N* = 710) and study 2 (*N* = 409), and subsequently combining the two studies to verify the differential validity of the scale by two industries. With two groups of frontline workers from two industries respectively, this allows for preliminary evidence of the applicability of the newly developed scale when used across fields. DeVellis argues that sample size should be as large as possible for representations of factor stability, but most studies of scale development have not adopted a consistent standard, although more researchers accept that sample size to item ratio is >10:1 (53, 59).

The scale contains 5 domains and 21 items, all of which can be grouped into the original domain. However, there were 6 domains in original generation, and we removed “Co-worker Support” because all items in this domain were poorly relevant to the total. In addition, the other five domains can express the relationship among co-workers to some extent, because some items use “we” as the first subject, referring to my common perception with colleagues. In the prevention of occupational diseases, organizational ownership and support are crucial. The mutual support among co-workers may be more closely related to lifestyle. However, as we learned to some extent during our research, interactions among workers are not easily captured in terms of occupational health protection because all of them are only expected to comply with the organization's management.

### Limitations

(1) OHC should be widely available in all workplaces in China's manufacturing industry. Although respondents in this research belong to the high occupational health risk group due to exposing to occupational hazards, it is difficult to confirm whether this scale is representative. In addition, even as copper producers, large producers have a long-term policy on OHS strategies, including developing culture, whereas small and medium enterprises (SMEs) paid attention to solving *ad-hoc* and short-term issues (60). Company size could lead to differences in the way employees and employer approach occupational health culture. Therefore, we need to include more industries and companies in next work and keep improving the OHCS scale.

(2) The respondents were entirely male because the construction and mining industries, especially the front-line production workers, are male-dominated. The more male-dominated occupational groups, the lower the scores of health literacy in the workplace (61). However, this may yield bias in the reliability and validity of scale. Attributable burden for occupational risks in China was higher in males than in females (4), especially occupational pneumoconiosis (62). Several research have found no significant gender differences in workplace health culture score, and differences exist in occupational health attitudes and occupational health protective behaviors (27, 46, 63). We believe that women workers as a large group of workers should be taken into consideration next, especially in the light industry.

(3) There is no reliable assessment tool and data for assessing the level of contamination in the workplace environment in China. The relationship between the effects of noise and illumination on miners' OHCS was unexpected. This is because noise and illumination have a significant effect on workers' physical and mental health (64–66). It is unclear whether the method is biased or this is the real situation. Within the occupational risk perception of workers, the risk tolerance is higher due to the poor health literacy (67, 68). We need to develop a more accurate tool to assess the physical environment and workers' attitudes toward occupational health risks.

### Suggestions

(1) For construction workers and miners, OHCS scores are lower from the ages of 31–40, and occupational health training should be enhanced for workers in this age group, who are in their prime and easily overlook potential health risks. To improve their occupational health literacy level through education and cultural inculcation during the important period of occupational disease prevention. In addition, as age and length of work increase, OHCS scores do not improve as significantly as expected, so we suggest that occupational health education should be provided continuously to workers on an ongoing basis, not just at the enters the duty initial period.

(2) Variety of job may have a great impact on OHCS perception scores due to different personal factors and the level of occupational hazard contamination in the workplace. Precise targeted education interventions to occupational health can be provided to workers according to their job type and occupational hazards they are exposed to. For some hazards where the perception of occupational hazards is not obvious, such as noise and illumination, improve workers' risk perception and prevention for them.

(3) The results of the study showed that a significant negative correlation between the respirable dust concentration from individual sampler and the OHCS scores of miners (−0.469^**^). The portable individual dust sampler can measure respirable dust concentrations that workers are exposed to more accurately, as they can be responded to laterally through OHCS. Therefore, strengthening individual occupational hazards monitoring, by combining objective individual exposure data and subjective individual perception, can more accurately measure the exposure of workers, while data from samplers installed at fixed measurement points in workplace can be used as a reference for exposure values.

(4) The Chinese government is implementing a program “Healthy Enterprise Development,” in which the development of a workplace health culture is a priority, and the protection of workers' occupational health should be a primary objective. OHC is a long-term project that needs to be better integrated with the organizational culture. The regulatory authorities should form policies and regulations that can be relied on, and develop specific health culture performance according to the characteristics of the industry. This scale is a measurement tool for individual-level occupational health culture perception score, and should be used in conjunction with organizational-level OHC to form a comprehensive organizational OHC evaluation system.

## Conclusion

(1) This paper initiated the occupational health culture in China and the scale model's confirmation factor analysis yielded acceptable results, indicating great reliability and validity of the scale. Preliminary applications were conducted in two industries with high occupational health risks, with differential analysis of the OHCS scores of miners and construction workers, which validated the applicability of the scale. The five domains of the OHCS scale and the design of the 21 items a, are in accordance with the Chinese cultural background and the way of occupational health management, which to some extent indicates the generality and extensibility of this scale.

(2) In this study, the differences in OHCS scores at the individual level were analyzed in terms of personal factors (age, education level, month income, and length of work), industry, type of job, and degree of contamination from occupational hazards at the job, respectively. The results of individual respiratory dust sampling by miners showed that individual respiratory dust exposure of miners had the largest negative correlation effect on OHCS scores, compared to the total dust and respiratory dust concentrations at fixed monitoring sites in the workplace. This result provides evidence for mining companies to strengthen individual miners' respiratory dust protection and for developing OHC in different types of companies.

(3) This scale is designed to better respond to and assess OHC aiming to providing the driving force for occupational health promotion in China, which is also the demand of China's large workforce and the government's goals. By developing an OHC, it can benefit more workers, which is conducive to the implementation of the Chinese government's “Occupational Health Protection Initiative” and the promotion of the “Healthy Enterprise Development.”

## Data availability statement

The original contributions presented in the study are included in the article/Supplementary material, further inquiries can be directed to the corresponding author.

## Author contributions

XY initiated the conceptualization, methodology, investigation, and writing—original draft of the manuscript. XZ was involved in the investigation software, validation, and data curation. YW took part in investigation and data curation. RT contributed to conceptualization, writing—review and editing supervision, and funding acquisition of the manuscript. All authors had full access to all the data in the study and had final responsibility for the decision to submit for publication.

## Funding

This work was supported by grant (No. 52074302) from the National Natural Science Foundation of China and by grant (No. 8212015) from the Natural Science Foundation of Beijing.

## Conflict of interest

The authors declare that the research was conducted in the absence of any commercial or financial relationships that could be construed as a potential conflict of interest.

## Publisher's note

All claims expressed in this article are solely those of the authors and do not necessarily represent those of their affiliated organizations, or those of the publisher, the editors and the reviewers. Any product that may be evaluated in this article, or claim that may be made by its manufacturer, is not guaranteed or endorsed by the publisher.

## References

[1] World Health Organization. Healthy Workplaces: A Model for Action. WHO (2010). Available online at: https://wwwwhoint/publications/i/item/healthy-workplaces-a-model-for-action (accessed April 21, 2022).

[2] Tong R Cheng M Zhang L Liu M Yang X Li X. The construction dust-induced occupational health risk using Monte-Carlo simulation. J Clean Prod. (2018) 184:598–608. 10.1016/j.jclepro.2018.02.286

[3] GBD 2016 Occupational Chronic Respiratory Risk Factors Collaborators. Global and regional burden of chronic respiratory disease in 2016 arising from non-infectious airborne occupational exposures: a systematic analysis for the global burden of disease study 2016. Occup Environ Med. (2020) 77:142–50. 10.1136/oemed-2019-106013 32054818 PMC7035690

[4] Li J Yin P Wang H Zeng X Zhang X Wang L. The disease burden attributable to 18 occupational risks in China: an analysis for the global burden of disease study 2017. Environ Health. (2020) 19:21. 10.1186/s12940-020-00577-y 32075644 PMC7031932

[5] Jones W Gibb A Haslam R Dainty A. Work-related ill-health in construction: the importance of scope, ownership and understanding. Saf Sci. (2019) 120:538–50. 10.1016/j.ssci.2019.07.038

[6] Xian W Han B Xia L Ma Y Xu H Zhang L. Focusing on the premature death of redeployed miners in China: an analysis of cause-of-death information from non-communicable diseases. Glob Health. (2019) 15:7. 10.1186/s12992-019-0450-5 30670067 PMC6341550

[7] Robson LS Clarke JA Cullen K Bielecky A Severin C Bigelow PL. The effectiveness of occupational health and safety management system interventions: a systematic review. Saf Sci. (2007) 45:329–53. 10.1016/j.ssci.2006.07.00319806275

[8] da Silva SLC Amaral FG. Critical factors of success and barriers to the implementation of occupational health and safety management systems: a systematic review of literature. Saf Sci. (2019) 117:123–32. 10.1016/j.ssci.2019.03.026

[9] Los FS de Boer AGEM van der Molen HF Hulshof CTJ. The implementation of workers' health surveillance by occupational physicians: a survey study. J Occup Environ Med. (2019) 61:e497–502. 10.1097/JOM.0000000000001740 31626069

[10] Wyatt KM Brand S Ashby-Pepper J Abraham J Fleming LE. Understanding how healthy workplaces are created: implications for developing a national health service healthy workplace program. Int J Health Serv. (2015) 45:161–85. 10.2190/HS.45.1.m 26460455

[11] O'Donnell M. Does workplace health promotion work or not? Are you sure you really want to know the truth? Am J Health Promot. (2013) 28:iv–vi. 10.4278/ajhp.28.1.iv 24000969

[12] Calderon A Harris JD Kirsch PA. Health interventions used by major resource companies operating in Colombia. Resour Policy. (2016) 47:187–97. 10.1016/j.resourpol.2015.02.003

[13] Safeer R Allen J. Defining a culture of health in the workplace. J Occup Environ Med. (2019) 61:863–7. 10.1097/JOM.0000000000001684 31348414

[14] Waterworth P Pescud M Chappell S Davies C Roche D Shilton T. Culture, management and finances as key aspects for healthy workplace initiatives. Health Promot Int. (2018) 33:162–72. 10.1093/heapro/daw068 27543456

[15] O'Toole M. The relationship between employees' perceptions of safety and organizational culture. J Saf Res. (2002) 33:231. 10.1016/S0022-4375(02)00014-2 12216448

[16] Kashan AJ Wiewiora A Mohannak K. Unpacking organisational culture for innovation in Australian mining industry. Resour Policy. (2021) 73:102149. 10.1016/j.resourpol.2021.102149

[17] Terry PE Seaverson ELD Grossmeier J Anderson DR. Association between nine quality components and superior worksite health management program results. J Occup Environ Med. (2008) 50:633–41. 10.1097/JOM.0b013e31817e7c1c 18545090

[18] Webb KM Krick D Co H Study Committee Definitions Workgroup. The Art of health promotion: linking research to practice. Am J Health Promot. (2017) 31:515–29. 10.1177/0890117117735957 29065714

[19] Schein E. Organizational culture. Am Psychol. (1990) 45:109–19. 10.1037/0003-066X.45.2.109

[20] Schein H. Organizational Culture and Leadership. Hoboken, NJ: John Wiley & Sons (2017)

[21] Tarique I Briscoe D Schuler R. International Human Resource Management: Policies and Practices for Multinational Enterprises. New York, NY: Routledge (2016).

[22] Baek P Chang J Kim T. Organizational culture now and going forward. J Organ Chang Manage. (2019) 32:650–68. 10.1108/JOCM-05-2018-0121

[23] Freiling J Fichtner H. Organizational culture as the glue between people and organization: a competence-based view on learning and competence building. Z Personalforsch. (2010) 24:152–72. 10.1177/239700221002400204

[24] Reiman T Oedewald P. Assessment of complex sociotechnical systems-Theoretical issues concerning the use of organizational culture and organizational core task concepts. Saf Sci. (2007) 45:745–69. 10.1016/j.ssci.2006.07.010

[25] Taylor WC Suminski RR Das BM Paxton RJ Craig DW. Organizational culture and implications for workplace interventions to reduce sitting time among office-based workers: a systematic review. Front Public Health. (2018) 6:263. 10.3389/fpubh.2018.00263 30320051 PMC6165892

[26] Allen J. The role of subcultures in wellness initiatives. J Health Promot. (2018) 32:1815–6. 10.1177/0890117118804149a 30350729

[27] Golaszewski T Hoebbel C Crossley J Foley G Dorn J. The reliability and validity of an organizational health culture audit. Am J Health Stud. (2008) 23:116–23.

[28] Allen J. Building supportive cultural environments. In: O'Donnell MP, editor. Health Promotion in the Workplace. New York, NY: Delmar Publishers Inc. (2002). p. 202–17.

[29] Hoebbel C Golaszewski T Swanson M Dorn J. Associations between the worksite environment and perceived health culture. Am J Health Promot. (2012) 26:301–4. 10.4278/ajhp.101118-ARB-381 22548425

[30] Aldana S Anderson D Adams T Whitmer RW Merrill R George V. A review of the knowledge base on healthy worksite culture. J Occup Environ Med. (2012) 54:414–9. 10.1097/JOM.0b013e31824be25f 22446571

[31] Allen J. Closing commentary: what I have learned about creating wellness cultures. Am J Health Promot. (2017) 31:527–9.

[32] Kent K Goetzel RZ Roemer EC Prasad A Freundlich N. Promoting healthy workplaces by building cultures of health and applying strategic communications. J Occup Environ Med. (2016) 58:114–22. 10.1097/JOM.0000000000000629 26849254

[33] Kwon Y Marzec ML Edington DW. Development and validity of a scale to measure workplace culture of health. J Occup Environ Med. (2015) 57:571–7. 10.1097/JOM.0000000000000409 25738947

[34] Chang Y Tsai F Kuo C Yeh C Chen R. Exploring and developing the workplace health culture scale in Taiwan. Front Public Health. (2019) 7:397. 10.3389/fpubh.2019.00397 31998678 PMC6965151

[35] Wang B Wu C. Safety culture development, research, and implementation in China: an overview. Prog Nucl Energy. (2019) 110:289–300. 10.1016/j.pnucene.2018.10.00235281902

[36] Fang D Wu H. Development of a safety culture interaction (SCI) model for construction projects. Saf Sci. (2013) 57:138–49. 10.1016/j.ssci.2013.02.003

[37] Guldenmund FW. The nature of safety culture: a review of theory and research. Saf Sci. (2000) 34:215–57. 10.1016/S0925-7535(00)00014-X

[38] Cooper MD. Towards a model of safety culture. Saf Sci. (2000) 36:111–36. 10.1016/S0925-7535(00)00035-7

[39] Govender U van Eck G Genc B. An integrated 4Cs safety framework for the diamond industry of Southern Africa. Resour Policy. (2022) 77:102774. 10.1016/j.resourpol.2022.102774

[40] Martyka J Lebecki K. Safety culture in high-risk industries. Int J Occup Saf Ergon. (2014) 20:561–72. 10.1080/10803548.2014.11077076 25513792

[41] Ismail S Ramli A Aziz H. Influencing factors on safety culture in mining industry: a systematic literature review approach. Resour Policy. (2021) 74:102250. 10.1016/j.resourpol.2021.102250

[42] Zhang J Fu J Hao H Fu G Nie F Zhang W. Root causes of coal mine accidents: characteristics of safety culture deficiencies based on accident statistics. Process Saf Environ Protect. (2020) 136:78–91. 10.1016/j.psep.2020.01.024

[43] Fu G. Safety Management-a Behavior-Based Approach to Accident Prevention. Beijing: Science Press (2013).

[44] Stewart JM. Managing for World Class Safety. New York, NY: Wiley-Interscience Publication (2002). 10.1002/9781118591444

[45] Chang Y Tsai F Yeh C Chen R. From cognition to behavior: associations of workplace health culture and workplace health promotion performance with personal healthy lifestyles. Front Public Health. (2021) 9:745846. 10.3389/fpubh.2021.745846 34820351 PMC8606586

[46] Jia Y Gao J Dai J Zheng P Fu H. Associations between health culture, health behaviors, and health-related outcomes: a cross-sectional study. PLoS ONE. (2017) 12:e0178644. 10.1371/journal.pone.0178644 28746400 PMC5528893

[47] Jia Y Fu H Gao J Dai J Zheng P. The roles of health culture and physical environment in workplace health promotion: a two-year prospective intervention study in China. BMC Public Health. (2018) 18:457. 10.1186/s12889-018-5361-5 29621986 PMC5887264

[48] Jia Y Wu X Li G Wang Y Fu H. Development and validation of workplace health culture scale and organizational health scale. Fudan Univer J Med Sci. (2015) 42:84–9. 10.1037/t67419-000

[49] Jiang W Fu G Liang C Han W. Study on quantitative measurement result of safety culture. Saf Sci. (2020) 128:104751. 10.1016/j.ssci.2020.104751

[50] Flynn JP Gascon G Doyle S Matson Koffman D Saringer C Grossmeier J. Supporting a culture of health in the workplace: a review of evidence-based elements. Am J Health Promot. (2018) 32:1755–88. 10.1177/0890117118761887 29806469

[51] Payne J Cluff L Lang J Matson-Koffman D Morgan-Lopez A. Elements of a workplace culture of health, perceived organizational support for health, lifestyle risk. Am J Health Promot. (2018) 32:1555–67. 10.1177/0890117118758235 29529865 PMC6106858

[52] Boateng GO Neilands TB Frongillo EA Melgar-Quinonez HR Young SL. Best practices for developing and validating scales for health, social, and behavioral research: a primer. Front Public Health. (2018) 6:149. 10.3389/fpubh.2018.00149 29942800 PMC6004510

[53] Morgado FFR Meireles JFF Neves CM Amaral ACS Ferreira MEC. Scale development: ten main limitations and recommendations to improve future research practices. Psicol Reflex Crit. (2017) 30:3. 10.1186/s41155-016-0057-1 32025957 PMC6966966

[54] Wolfe WS Frongillo EA. Building household food-security measurement tools from the ground up. Food Nutr Bull. (2001) 22:5–12. 10.1177/156482650102200102

[55] Mohandes SR Zhang X. Developing a holistic occupational health and safety risk assessment model: an application to a case of sustainable construction project. J Clean Prod. (2021) 291:125934. 10.1016/j.jclepro.2021.125934

[56] Zhang Q Zhou G Qian X Yuan M Sun Y Wang D. Diffuse pollution characteristics of respirable dust in fully-mechanized mining face under various velocities based on CFD investigation. J Clean Prod. (2018) 184:239–50. 10.1016/j.jclepro.2018.02.230

[57] Fabrigar LR Wegener DT MacCallum RC Strahan EJ. Evaluating the use of exploratory factor analysis in psychological research. Psychol Methods. (1999) 4:272–99. 10.1037/1082-989X.4.3.27219609833

[58] Huang X Chen H Long R Li S. Development and validation of the quality of life scale for Chinese coal miners with pneumoconiosis (QOL-CMP): measurement method and empirical study. J Clean Prod. (2019) 232:1062–75. 10.1016/j.jclepro.2019.05.398

[59] DeVellis RF. Scale Development: Theory and Applications. Newbury Park, CA: Sage Publications (2003).

[60] Fordham AE Robinson GM Blackwell BD. Corporate social responsibility in resource companies – opportunities for developing positive benefifits and lasting legacies. Resour Policy. (2017) 52:366–76. 10.1016/j.resourpol.2017.04.009

[61] Milner A Shields M Scovelle AJ Sutherland G King TL. Health literacy in male-dominated occupations. Am J Mens Health. (2020) 14:1557988320954022. 10.1177/1557988320954022 33054500 PMC7570794

[62] Liu L. China's dusty lung crisis: rural-urban health inequity as social and spatial injustice. Soc Sci Med. (2019) 233:218–28. 10.1016/j.socscimed.2019.05.033 31229908

[63] Lim W Ma S Heng D Bhalla V Chew SK. Gender, ethnicity, health behaviour & self-rated health in Singapore. BMC Public Health. (2007) 7:184. 10.1186/1471-2458-7-184 17655774 PMC1976324

[64] Bao J Song X Li Y Bai Y Zhou Q. Effect of lighting illuminance and colour temperature on mental workload in an office setting. Sci Rep. (2021) 11:15284. 10.1038/s41598-021-94795-0 34315983 PMC8316362

[65] Li B Wang E Shang Z Xu G Ali M Wang H. Quantification study of working fatigue state affected by coal mine noise exposure based on fuzzy comprehensive evaluation. Saf Sci. (2022) 146:105577. 10.1016/j.ssci.2021.105577

[66] Zhao S He D Zhang H Hou T Yang C Ding W. Health study of 11,800 workers under occupational noise in Xinjiang. BMC Public Health. (2021) 21:460. 10.1186/s12889-021-10496-3 33676457 PMC7937221

[67] Eiter BM Bellanca JL. Identify the influence of risk attitude, work experience, and safety training on hazard recognition in mining. Min Metall Explor. (2020) 37:1931–9. 10.1007/s42461-020-00293-8 34734163 PMC8563016

[68] Le Berre S Bretesché S. Having a high-risk job: uranium miners' perception of occupational risk in France. Extract Indust Soc. (2020) 7:568–75. 10.1016/j.exis.2019.11.011

