# Supplementary material for: Development of occupational health culture scale: A study based on miners and construction workers
Source: Front Public Health. 2022 Aug 22;10:992515. doi: 10.3389/fpubh.2022.992515 (PMC9441892; doi:10.3389/fpubh.2022.992515)
Supplement: Supplementary file 1 [file Data_Sheet_1.docx]

# Table S1 Elements of workplace health culture and safety culture

| **Culture** | **Elements** | | | |
| --- | --- | --- | --- | --- |
| Workplace health culture by HERO | - Communication - Employee involvement and empowerment - Executive leadership - External community connections and altruism - Internal customer orientation - Metrics and measurement | - Modeling - Norms - Organizational Leadership - Organizational resource allocation and commitment - Orientation and first impressions - Peer support | - Policies and procedures - Positive outlook - Push back - Recruitment and selection - Relationship Development - Rewards and recognition | - Sense of community - Shared vision and mission - Shared values - Supportive built environment - Traditions and symbols - Training and learning |
| Safety culture by Fu | - Relative importance of safety - Preventable extent of deaths and injuries - Safety creates economic benefits - Degree of safety into enterprise management - Safety depends mainly on safety awareness - Responsibility of work safety - Awareness of safety input - Role of safety regulations | - Safety values formation level - Degree of leaders’ responsibility - Understanding of the role of the safety sector - Degree of employee’s participation in safety - Level of safety training needs - Degree of the department’s safety responsibility - Effects of community safety - Function of safety management system | - Safety council demands - Formation way of safety system - Consistent implementation of safety systems - Types of investigated accidents - Types of safety check - Caring for injured workers - Amateur safety management - Treatment of safety performance | - Facilities satisfaction - Mastering of Safety performance - Safety performance and human resources - Safety management of subsidiary and co-firms - Function of safety organization - Work of safety department - Overall safety expectations - Emergency response capabilities |

# Table S2 Definitions of the occupational health culture scale

| **Domains** | **Definition** |
| --- | --- |
| Leadership support | Represent the vision of the organization and can decide and implement the allocation of occupational health resources. Middle and grassroots leaders manage workers directly, the transmit occupational health information, and support employees' protection behaviors of occupational health and usage of resource. |
| Coworker support | Colleagues at work provide each other with health-related knowledge, experience, emotion, socialization, encouragement while maintaining altruism and common will. |
| Values | It can be divided into shared values and personal values. Shared values are employees' consistent views on the importance of occupational health-related issues; personal values are individuals' beliefs and attitudes toward occupational health. |
| Policy and norms | On the basis of OHSMS framework, by internal policies reinforce and implementation of occupational health-related actions, employees form a consistent, normative way of behavior that requires management intervention to shape and reinforce it. |
| Employee involvement | Employees are involved to some degree in making decisions about production practices on worksite, advocating for more health-friendly production solutions. They are also empowered and granted autonomy to promote healthier, safer, and more efficient engagement in their work. |
| Physical environment | The existences of occupational hazards and physical structures in the workplace environment. There are two main categories to consider: occupational health hazards, such as dust; and design of ergonomics. This is determined by the position and industry. |

# Table S3 The content and code of 57 items

| **Code** | **Items**  (1 – strongly disagree, 5 - strongly agree) |
| --- | --- |
| **Leader support (LS)** |  |
| LS1 | I believe in the leadership's commitment to my occupational health protection. |
| LS2 | I believe leaders often mention health protection of employee in the course of speeches. |
| LS3 | I believe company leaders are the main responsible for our occupational health. |
| LS4 | I believe leaders or companies value the health and wellness of their employees. |
| LS5 | I believe that the company's occupational health management is closely related to the principal leaders of company. |
| LS6 | I believe the foreman will report the contamination of hazards to higher leadership. |
| LS7 | I think my foreman will inform me if I had a higher occupational health risk. |
| LS8 | My foreman will always remind me to check or change my protective equipment. |
| LS9 | My foreman pays close attention to personal protection on site. |
| LS10 | My foreman is very concerned about the contamination of hazards in the workplace environment. |
| LS11 | My foreman finish managing occupational health with care. |
| **Coworker support (CS)** |  |
| CS1 | I adopt cleaner production methods, which also benefit the occupational health of all coworkers in the workplace. |
| CS2 | My coworkers will remind me to check or change protective equipment. |
| CS3 | I am willing to consult a health physician with a group of coworkers. |
| CS4 | My coworkers and I will be happy to discuss occupational health screening indicators. |
| CS5 | My coworkers and I will care about wellness each other. |
| CS6 | My coworkers will often give me advice on occupational health protection. |
| CS7 | I think that my coworkers will help me to do some work to remove occupational hazards. |
| CS8 | I think that my coworkers will support me to take sick leave due to ill health. |
| CS9 | I can stop and advise my coworkers who are producing large amounts of pollutants. |
| **Value (V)** |  |
| V1 | I think it is important to put your own safety and health first. |
| V2 | I think that the company gives us reasonable occupational health protection. |
| V3 | I believe that the company has the ability to protect our occupational health. |
| V4 | I believe that protecting occupational health is a way to increase the long-term profitability of a company. |
| V5 | Company references to employee wellness in business objectives or organizational mission statements. |
| V6 | I think the company improve our workplace environment is for our occupational health, let us work healthy. |
| V7 | I believe that work-related diseases can be prevented. |
| V8 | I believe that occupational health is more important than earning money. |
| **Physical environment (PE)** |  |
| PE1 | I can perceive the existence of occupational hazards in the workplace environment. |
| PE2 | I feel that my workplace environment makes me acceptable. |
| PE3 | I think I adapt easily to my work environment. |
| PE4 | I feel that the protection system is effective in the workplace environment. |
| PE5 | I can see signs in the workplace about health hazards. |
| PE6 | I think it is reasonable to place equipment in the workplace. |
| PE7 | We regularly clean and inspect occupational health hazards. |
| PE8 | We think that companies are constantly investing in improving our workplace environment. |
| **Policy and norms (PN)** |  |
| PN1 | I think that the protective equipment used by the company according to the standard. |
| PN2 | I think OHS specialists play a role in terms of wellness. |
| PN3 | I believe that the company's improvement of our workplace environment is not a response to supervision. |
| PN4 | I think that the medical examination set by the company for us is reasonable. |
| PN5 | I think that the company will transfer my position according to my health condition. |
| PN6^a^ | I believe that it is the company's social responsibility to protect occupational health. |
| PN7 | I believe that the company is willing to give us industrial injury insurance. |
| PN8 | I think that the company will advise us to purchase other commercial medical insurance. |
| PN9 | I know how to respond to occupational health emergencies. |
| PN10 | I believe the company will carefully analyze and inform us of the results of the occupational medical examination. |
| PN11 | We will regularly check or replace protective equipment. |
| PN12 | The company regularly checks the contamination of hazards in the environment. |
| **Employee involvement (EI)** |  |
| EI1 | I am willing to discuss cleaner production options with the foreman. |
| EI2 | I will discuss my occupational health issues with my company's health consultant or health advisor. |
| EI3 | I like to participate in the health activities organized by the company. |
| EI4 | I had the opportunity to give some advice to the leaders or supervisors about occupational health. |
| EI5 | The company often trains us on how to prevent work-related diseases. |
| EI6 | The foreman usually informs us the occupational hazards in the workplace and discuss the solutions with us. |
| EI7 | The foreman will seriously consider our comments on the cleaner production program. |
| EI8 | I participate in the occupational disease prevention and control programs in my work team. |
| EI9 | Our work team will listen to our views when developing occupational health policies. |

# Table S4 Reference description and criterion for the fitness index of the structural equation model

| **Index type** | **Index** | **Reference** **criterion** | **Ideal criterion** |
| --- | --- | --- | --- |
| Absolute fitting index | Standardized root mean square residual  (SRMR) | > 0 | < 0.08, <0.05 is better |
|  | Chi-square / degree of freedom  (χ^2^ /df) | > 0 | <5，<3 is better |
|  | Goodness-of- fit index  (GFI) | 0~1 | > 0.85，>0.9 is better |
|  | Adjusted for the model's degrees of freedom  (AGFI) | 0~1 | >0.85，>0.9 is better |
|  | Root mean square error of approximation  (RMSEA) | > 0 | <0.1，<0.05 is better |
| Relative fit index | Normal fit index  (NFI) | 0~1 | >0.85，>0.9 is better |
|  | Incremental fit index  (IFI) | 0~1 | >0.85，>0.9 is better |
|  | Tucker-Lewis Index  (TLI) | 0~1 | >0.85，>0.9 is better |
|  | Comparative fit index  (CFI) | 0~1 | >0.85，>0.9 is better |

# Table S5The content and code of 46 items

| **Code** | **Items**  (1 – strongly disagree, 5 - strongly agree) |
| --- | --- |
| **Leader support (LS)** |  |
| LS1 | I believe in the leadership's commitment to my occupational health protection. |
| LS3 | I believe company leaders are the main responsible for our occupational health. |
| LS4 | I believe leaders or companies value the health and wellness of their employees. |
| LS6 | I believe the foreman will report the contamination of hazards to higher leadership. |
| LS8 | My foreman will always remind me to check or change my protective equipment. |
| LS9 | My foreman pays close attention to personal protection on site. |
| LS10 | My foreman is very concerned about the contamination of hazards in the workplace environment. |
| LS11 | My foreman finish managing occupational health with care. |
| **Coworker support (CS)** |  |
| CS1 | I adopt cleaner production methods, which also benefit the occupational health of all coworkers in the workplace. |
| CS2 | My coworkers will remind me to check or change protective equipment. |
| CS3 | I am willing to consult a health physician with a group of coworkers. |
| CS4 | My coworkers and I will be happy to discuss occupational health screening indicators. |
| CS6 | My coworkers will often give me advice on occupational health protection. |
| CS7 | I think that my coworkers will help me to do some work to remove occupational hazards. |
| CS9 | I can stop and advise my coworkers who are producing large amounts of pollutants. |
| **Value (V)** |  |
| V1 | I think it is important to put your own safety and health first. |
| V2 | I think that the company gives us reasonable occupational health protection. |
| V4 | I believe that protecting occupational health is a way to increase the long-term profitability of a company. |
| V5 | Company references to employee wellness in business objectives or organizational mission statements. |
| V6 | I think the company improve our workplace environment is for our occupational health, let us work healthy. |
| V7 | I believe that work-related diseases can be prevented. |
| V8 | I believe that occupational health is more important than earning money. |
| **Physical environment (PE)** |  |
| PE1 | I can perceive the existence of occupational hazards in the workplace environment. |
| PE2 | I feel that my workplace environment makes me acceptable. |
| PE4 | I feel that the protection system is effective in the workplace environment. |
| PE5 | I can see signs in the workplace about health hazards. |
| PE6 | I think it is reasonable to place equipment in the workplace. |
| PE7 | We regularly clean and inspect occupational health hazards. |
| PE8 | We think that companies are constantly investing in improving our workplace environment. |
| **Policy and norms (PN)** |  |
| PN2 | I think OHS specialists play a role in terms of wellness. |
| PN3 | I believe that the company's improvement of our workplace environment is not a response to supervision. |
| PN4 | I think that the medical examination set by the company for us is reasonable. |
| PN5 | I think that the company will transfer my position according to my health condition. |
| PN7 | I believe that the company is willing to give us industrial injury insurance. |
| PN8 | I think that the company will advise us to purchase other commercial medical insurance. |
| PN9 | I know how to respond to occupational health emergencies. |
| PN11 | We will regularly check or replace protective equipment. |
| PN12 | The company regularly checks the contamination of hazards in the environment. |
| **Employee involvement (EI)** |  |
| EI1 | I am willing to discuss cleaner production options with the foreman. |
| EI3 | I like to participate in the health activities organized by the company. |
| EI4 | I had the opportunity to give some advice to the leaders or supervisors about occupational health. |
| EI5 | The company often trains us on how to prevent work-related diseases. |
| EI6 | The foreman usually informs us the occupational hazards in the workplace and discuss the solutions with us. |
| EI7 | The foreman will seriously consider our comments on the cleaner production program. |
| EI8 | I participate in the occupational disease prevention and control programs in my work team. |
| EI9 | Our work team will listen to our views when developing occupational health policies. |

# Table S6 The content and code of 24 items

| **Code** | **Items**  (1 – strongly disagree, 5 - strongly agree) |
| --- | --- |
| **Leader support (LS)** |  |
| LS3 | I believe company leaders are the main responsible for our occupational health. |
| LS4 | I believe leaders or companies value the health and wellness of their employees. |
| LS8 | My foreman will always remind me to check or change my protective equipment. |
| LS9 | My foreman pays close attention to personal protection on site. |
| LS10 | My foreman is very concerned about the contamination of hazards in the workplace environment. |
| **Value (V)** |  |
| V1 | I think it is important to put your own safety and health first. |
| V4 | I believe that protecting occupational health is a way to increase the long-term profitability of a company. |
| V5# | Company references to employee wellness in business objectives or organizational mission statements. |
| V7 | I believe that work-related diseases can be prevented. |
| **Physical environment (PE)** |  |
| PE1 | I can perceive the existence of occupational hazards in the workplace environment. |
| PE4 | I feel that the protection system is effective in the workplace environment. |
| PE5# | I can see signs in the workplace about health hazards. |
| PE6 | I think it is reasonable to place equipment in the workplace. |
| PE7 | We regularly clean and inspect occupational health hazards. |
| **Policy and norms (PN)** |  |
| PN3# | I believe that the company's improvement of our workplace environment is not a response to supervision. |
| PN4 | I think that the medical examination set by the company for us is reasonable. |
| PN5 | I think that the company will transfer my position according to my health condition. |
| PN8 | I think that the company will advise us to purchase other commercial medical insurance. |
| PN9 | I know how to respond to occupational health emergencies. |
| PN11 | We will regularly check or replace protective equipment. |
| **Employee involvement (EI)** |  |
| EI5 | The company often trains us on how to prevent work-related diseases. |
| EI6 | The foreman usually informs us the occupational hazards in the workplace and discuss the solutions with us. |
| EI7 | The foreman will seriously consider our comments on the cleaner production program. |
| EI9 | Our work team will listen to our views when developing occupational health policies. |

# Item deleted according to the modification index (M.I.)

# Table S7 Factor loading results for the 21 items in study 2

| **Domains** | **Items** | **Corrected**  **item-to-total**  **correlation** | **Cronbach’s α** | **Factor loadings** | | | | | **CR** | **AVE** | **Total explained**  **variance (%)** |
| --- | --- | --- | --- | --- | --- | --- | --- | --- | --- | --- | --- |
|  |  |  |  | 1 | 2 | 3 | 4 | 5 |  |  |  |
| Leadership support | LS3 | 0.720 | 0.95 | 0.882 |  |  |  |  | 0.9235 | 0.7091 | 20.63 |
|  | LS4 | 0.720 |  | 0.876 |  |  |  |  |  |  |  |
|  | LS8 | 0.724 |  | 0.872 |  |  |  |  |  |  |  |
|  | LS9 | 0.725 |  | 0.884 |  |  |  |  |  |  |  |
|  | LS10 | 0.585 |  | 0.677 |  |  |  |  |  |  |  |
| Values | V1 | 0.688 | 0.959 |  | 0.853 |  |  |  | 0.8825 | 0.7146 | 19.62 |
|  | V4 | 0.706 |  |  | 0.847 |  |  |  |  |  |  |
|  | V7 | 0.716 |  |  | 0.836 |  |  |  |  |  |  |
| Policy and norms | PN4 | 0.755 | 0.948 |  |  | 0.785 |  |  | 0.9066 | 0.6603 | 14.68 |
|  | PN5 | 0.643 |  |  |  | 0.846 |  |  |  |  |  |
|  | PN8 | 0.719 |  |  |  | 0.831 |  |  |  |  |  |
|  | PN9 | 0.786 |  |  |  | 0.779 |  |  |  |  |  |
|  | PN11 | 0.743 |  |  |  | 0.82 |  |  |  |  |  |
| Employee involvement | EI5 | 0.577 | 0.882 |  |  |  | 0.69 |  | 0.8379 | 0.5643 | 14.11 |
|  | EI6 | 0.688 |  |  |  |  | 0.758 |  |  |  |  |
|  | EI7 | 0.692 |  |  |  |  | 0.794 |  |  |  |  |
|  | EI9 | 0.697 |  |  |  |  | 0.759 |  |  |  |  |
| Physical environment | PE1 | 0.588 | 0.888 |  |  |  |  | 0.808 | 0.8713 | 0.629 | 13.08 |
|  | PE4 | 0.563 |  |  |  |  |  | 0.827 |  |  |  |
|  | PE6 | 0.753 |  |  |  |  |  | 0.746 |  |  |  |
|  | PE7 | 0.491 |  |  |  |  |  | 0.789 |  |  |  |

# Table S8 Correlations between factor structures in study 2

|  | OHV | OHLS | OHPN | OHEI | OHPE |
| --- | --- | --- | --- | --- | --- |
| OHV | — |  |  |  |  |
| OHLS | 0.470*** | — |  |  |  |
| OHPN | 0.644*** | 0.585*** | — |  |  |
| OHEI | 0.599*** | 0.618*** | 0.63*** | — |  |
| OHPE | 0.577*** | 0.532*** | 0.578*** | 0.589*** | — |
| Square root of AVE | 0.845 | 0.842 | 0.813 | 0.915 | 0.793 |

Note：***P<0.001

# Table S9 Test for correlation between personal factors and OHCS scores

|  |  | **TAS** | **OHLS** | **OHV** | **OHPN** | **OHEI** | **OHPE** |
| --- | --- | --- | --- | --- | --- | --- | --- |
| **A: Age** |  |  |  |  |  |  |  |
|  | A1：18-30 | 3.79 ± 0.17 | 3.69 ± 0.39 | 4.60 ± 0.44 | 3.60 ± 0.40 | 3.75 ± 0.28 | 3.59 ± 0.41 |
|  | A2：31-40 | 3.23 ± 0.47 | 3.12 ± 0.59 | 3.90 ± 0.65 | 3.15 ± 0.65 | 3.22 ± 0.58 | 2.98 ± 0.61 |
|  | A3：41-50 | 3.33 ± 0.45 | 3.20 ± 0.53 | 4.02 ± 0.73 | 3.30 ± 0.56 | 3.25 ± 0.64 | 3.07 ± 0.61 |
|  | A4：51-60 | 3.35 ± 0.49 | 3.27 ± 0.57 | 3.92 ± 0.68 | 3.30 ± 0.56 | 3.14 ± 0.75 | 3.32 ± 0.51 |
|  | A5：> 60 | 3.60 ± 0.39 | 3.61 ± 0.43 | 4.11 ± 0.48 | 3.50 ± 0.59 | 3.30 ± 0.47 | 3.63 ± 0.65 |
| Levene Test (Sig.) |  | 0.000 | 0.019 | 0.009 | 0.049 | 0.000 | 0.629 |
| Welch test (Sig.) |  | 0.000 | 0.000 | 0.000 | 0.000 | 0.000 | 0.000 |
| Multiple comparisons  (* P <0.05) |  | A5>A4*; A5>A3*; A5>A2*; A3>A2*; A1>A2*; A1>A3*; A1>A4 | A5>A4*; A5>A3*; A5>A2*; A4>A2*;  A1>A2*; A1>A3*;  A1>A4*; | A1>A2*; A1>A3*;  A1>A4*; A1>A5*; | A5>A2*; A3>A2*;  A1>A2*; | A1>A2*; A1>A3*;  A1>A4*; A1>A5*; | A5>A3*; A5>A2*; A5>A2*; A4>A3*; A4>A2*; A1>A2*;  A1>A3*; |
| **B: Education level** |  |  |  |  |  |  |  |
|  | B1: Primary school or below | 2.90 ± 0.44 | 2.90 ± 0.80 | 3.33 ± 0.51 | 2.83 ± 0.72 | 2.75 ± 0.52 | 2.79 ± 0.53 |
|  | B2: Junior high school | 3.20 ± 0.45 | 3.11 ± 0.55 | 3.85 ± 0.64 | 3.14 ± 0.58 | 3.19 ± 0.66 | 2.94 ± 0.60 |
|  | B3: High school or technical secondary school | 3.32 ± 0.45 | 3.22 ± 0.53 | 3.99 ± 0.69 | 3.28 ± 0.57 | 3.20 ± 0.64 | 3.15 ± 0.60 |
|  | B4: Junior college | 3.63 ± 3.72 | 3.48 ± 0.53 | 4.34 ± 0.69 | 3.62 ± 0.50 | 3.42 ± 0.57 | 3.50 ± 0.46 |
|  | B5: Bachelor or above | 3.72 ± 0.20 | 3.78 ± 0.53 | 4.36 ± 0.50 | 3.51 ± 0.45 | 3.48 ± 0.48 | 3.66 ± 0.44 |
| Levene Test (Sig.) |  | 0.000 | 0.003 | 0.177 | 0.134 | 0.472 | 0.152 |
| Welch test (Sig.) |  | 0.000 | 0.000 | 0.000 | 0.000 | 0.000 | 0.000 |
| Multiple comparisons  (* P <0.05) |  | B5>B3*; B5>B2*; B5>B1*; B4>B3*, B4>B2*; B4>B1*; B3>B2*; | B5>B3*; B5>B2*; B5>B1*; B4>B3*, B4>B2*; B3>B2* | B5>B1*; B4>B3*, B4>B2*; B4>B1*; B3>B2*; B3>B1*; | B5>B1*; B4>B3*, B4>B2*; B4>B1*; B3>B2*; | B4>B3*, B4>B2*; B4>B1*; | B5>B3*; B5>B2*; B5>B1*; B4>B3*, B4>B2*; B4>B1*; B3>B2*; |
| **C: Month income** |  |  |  |  |  |  |  |
|  | C1: < 5000 | — | — | — | — | — | — |
|  | C2: 5000-7000 | 2.80 ± 0.18 | 2.77 ± 0.73 | 3.15 ± 0.38 | 3.03 ± 0.18 | 2.56 ± 0.38 | 2.54 ± 0.52 |
|  | C3: 7000-9000 | 3.10 ± 0.46 | 3.03 ± 0.58 | 3.72 ± 0.67 | 3.06 ± 0.57 | 2.93 ± 0.65 | 2.91 ± 0.58 |
|  | C4: 9000-11000 | 3.33 ± 0.44 | 3.21 ± 0.52 | 4.07 ± 0.71 | 3.28 ± 0.57 | 3.23 ± 0.59 | 3.11 ± 0.64 |
|  | C5: > 11000 | 3.49 ± 0.42 | 3.36 ± 0.52 | 4.10 ± 0.62 | 3.42 ± 0.59 | 3.46 ± 0.62 | 3.30 ± 0.54 |
| Levene Test (Sig.) |  | 0.000 | 0.005 | 0.000 | 0.000 | 0.027 | 0.037 |
| Welch test (Sig.) |  | 0.000 | 0.000 | 0.000 | 0.000 | 0.000 | 0.000 |
| Multiple comparisons  (* P <0.05) |  | C5>C4*; C5>C3*; C5>C2*; C4>C3*; C4>C2*; C3>C2*; | C5>C4*; C5>C3*; C4>C3*; | C5>C3*; C5>C2*; C4>C3*; C4>C2*; C3>C2*; | C5>C3*; C5>C2*; C4>C3*; C4>C2*; | C5>C4*; C5>C3*; C5>C2*; C4>C3*; C4>C2*; C3>C2*; | C5>C4*; C5>C3*; C5>C2*; C4>C3*; C4>C2*; |
| **D: Length of work** |  |  |  |  |  |  |  |
|  | D1: < 5 | 3.07 ± 0.47 | 2.97 ± 0.56 | 3.72 ± 0.66 | 2.94 ± 0.68 | 3.13 ± 0.56 | 2.80 ± 0.59 |
|  | D2: 6-10 | 3.33 ± 0.43 | 3.21 ± 0.54 | 3.99 ± 0.66 | 3.33 ± 0.54 | 3.29 ± 0.61 | 3.00 ± 0.60 |
|  | D3: 11-15 | 3.35 ± 0.47 | 3.25 ± 0.55 | 4.03 ± 0.70 | 3.27 ± 0.56 | 3.19 ± 0.68 | 3.24 ± 0.59 |
|  | D4: 16-20 | 3.48 ± 0.49 | 3.38 ± 0.54 | 4.05 ± 0.76 | 3.48 ± 0.61 | 3.26 ± 0.67 | 3.40 ± 0.50 |
|  | D5: > 20 | 4.00 ± 0.20 | 4.00 | 4.50 ± 0.71 | 3.90 ± 0.14 | 4.00 | 3.75 ± 0.35 |
| Levene Test (Sig.) |  | 0.039 | 0.083 | 0.047 | 0.032 | 0.000 | 0.528 |
| Welch test (Sig.) |  | 0.000 | 0.000 | 0.032 | 0.001 | 0.000 | 0.000 |
| Multiple comparisons  (* P <0.05) |  | D4>D1*; D3>D1* D2>D1*; | D4>D1*; D3>D1* D2>D1*; | D4>D1*; D3>D1* D2>D1*; | D4>D1*; D3>D1* D2>D1*; | D5>D4*; D5>D3* D5>D2*; D5>D1*; | D4>D2*; D4>D1* D3>D1*; D3>D2*; D2>D1*; |

# Table S10 Correlation test between workplace pollution and occupational health culture score among miners

| **Domain** | **Total dust** | | **Respiratory dust** | | **Individual respiratory dust** | | **Noise** | | **Illumination** | |
| --- | --- | --- | --- | --- | --- | --- | --- | --- | --- | --- |
|  | Two-tailed test  (Sig.) | Correlation | Two-tailed test  (Sig.) | Correlation | Two-tailed test  (Sig.) | Correlation | Two-tailed test  (Sig.) | Correlation | Two-tailed test  (Sig.) | Correlation |
| TAS | 0.034 | -0.100* | 0.018 | -0.112* | 0.000 | -0.469** | 0.000 | 0.212** | 0.000 | -0.369** |
| OHLS | 0.026 | -0.105* | 0.022 | -0.108* | 0.000 | -0.393** | 0.012 | 0.119* | 0.000 | -0.212** |
| OHV | 0.011 | -0.120* | 0.014 | -0.115* | 0.000 | -0.274** | 0.048 | 0.093* | 0.000 | -0.282** |
| OHPN | 0.533 | -0.029 | 0.427 | -0.037 | 0.000 | -0.375** | 0.000 | 0.256* | 0.000 | -0.287** |
| OHEI | 0.443 | 0.036 | 0.974 | -0.002 | 0.000 | -0.278** | 0.000 | 0.199** | 0.000 | -0.386** |
| OHPE | 0.002 | -0.144** | 0.004 | -0.136** | 0.000 | -0.370** | 0.031 | 0.102* | 0.000 | -0.199** |
